# Mediterranean diet and symptom severity in Sjogren’s syndrome

**DOI:** 10.3389/fmed.2026.1730536

**Published:** 2026-03-04

**Authors:** Bertha Maria Nassani, Hind Eid, Sarah El Tahech, Andrew el Alam, Celine Chaaya, Georges Maalouly

**Affiliations:** 1 Faculty of Medicine, Saint Joseph University of Beirut, Beirut, Lebanon; 2 Gilbert and Rose-Marie Chagoury School of Medicine, Lebanese American University, Byblos, Lebanon

**Keywords:** ESSPRI, fatigue, Mediterranean diet, OSDI score, Sjoegren’s syndrome

## Abstract

**Background:**

Sjogren’s syndrome (SS) is a multifaceted clinical condition characterized by various features, including ocular dryness (OD), dry mouth, arthralgia, and fatigue, which plays a substantial role in shaping the clinical presentation of the disease and has detrimental effects on quality of life. Recent research has acknowledged the advantages of the Mediterranean diet (MD) for its positive impact on various autoimmune diseases. This study aims to investigate the effect of Mediterranean diet on Sjogren disease using two validated scores: the Ocular Surface Disease Index (OSDI) score and the Eular Sjogren’s Syndrome Patient Reported Index (ESSPRI).

**Methods:**

This was a cross-sectional observational study of previously diagnosed patients of Sjogren Syndrome using the ACR EULAR 2016 criteria. Patients were recruited from the archives of a tertiary university hospital. Data were collected through a telephone questionnaire, including demographic and disease data, the Ocular Surface Disease Index (OSDI) score to evaluate the OD severity, the Mediterranean Diet Adherence Screener (MEDAS) score to determine adherence to the MD and the Eular Sjogren’s Syndrome Patient Reported Index (ESSPRI). The primary outcome of the study, the association between OSDI/ESSPRI and MEDAS scores, was evaluated using Spearman’s correlation coefficient.

**Results:**

The study comprised 75 patients with a mean age of 50.1 ± 13.3 years. There were 88% women among the patients. The median scores were 5.0 for the ESSPRI (IQR 3.0–6.7), 25.0 for the OSDI (IQR 10.4–40.6), and 8.0 for the MEDAS (IQR 4.5–11.0). The Spearman correlation analysis revealed a significant negative correlation between MEDAS and OSDI (ρ = −0.78, *p* < 0.001). None of the covariates were statistically significant in the ESSPRI model, whereas treatment exhibited 2 a positive correlation with OSDI (β = +6.44, 95% CI 0.84 to 12.03; *p* = 0.025), likely indicating confounding by indication.

**Conclusion:**

The study results suggest that higher adherence to a Mediterranean dietary pattern was consistently associated with lower symptom burden. This approach may serve as a complementary strategy with multiple health benefits, alongside conventional treatment options.

## 1 Introduction

Sjogren syndrome (SjS), a systemic autoimmune disease that affects 1–23 persons per 10000 inhabitants in European countries, presents with a wide spectrum of clinical manifestations and autoantibodies (1). SjS stands out as one of the most prevalent autoimmune diseases, displaying a broad systemic phenotype categorized into three distinct types: dry syndrome, systemic symptoms, and organ involvement. Its multifaceted origin arises from the intricate interplay between environmental, immunological, and genetic factors (2). The worldwide prevalence of primary SS is 0.06%, and a notable majority of cases occur in women, with a ratio of 9:1 (3, 4). It has an unusual age at diagnosis of around 60 years (between the 5th and the 7th decade of life) (5). In cases where SjS is diagnosed in younger patients (less than 35 years of age), presentation is frequently associated with fever, lymphadenopathy, and high disease activity (6). In recent years, the prevalence of autoimmune diseases has risen worldwide, largely influenced by lifestyle-related environmental factors. Growing evidence highlights the role of diet in modulating autoimmunity, with particular attention to the Mediterranean diet (MD). Characterized by high intake of fiber, vegetables, olive oil, and moderate consumption of fish, poultry, and wine, and with minimal processed foods and red meats, the MD provides nutrients with anti-inflammatory and antioxidant properties. Originating from Ancel Keys’ “Seven Countries Study” in 1960 and later recognized by UNESCO as Intangible Cultural Heritage, the MD has been associated with improved health outcomes, including reduced inflammation. Its potential relevance is underscored in autoimmune conditions such as Sjogren’s syndrome (SS), a chronic connective tissue disease characterized by lymphocytic infiltration of salivary and lacrimal glands and abnormal immune activation like systemic lupus erythematosus. While current findings suggest the MD may provide protective effects against autoimmunity, further well-designed randomized controlled trials are required to establish specific dietary recommendations. Nonetheless, encouraging adherence to the MD offers a promising, cost-effective strategy to complement conventional therapies (Symptomatic treatment of sicca symptomatology, and broad-spectrum immunosuppression for systemic disease) in reducing the burden of autoimmune diseases (7).

## 2 Materials and methods

### 2.1 Study population

This was a single center cross-sectional observational study. Participants were recruited from a tertiary university hospital, Ho^∧^tel-Dieu de France in Beirut, Lebanon. This study builds upon our previously published work examining the association between Mediterranean diet adherence and ocular dryness severity (OSDI) in Sjögren’s syndrome (8). While ocular outcomes are included here for completeness and confirmatory purposes, the primary objective of the present analysis is to evaluate the relationship between Mediterranean diet adherence and global patient-reported symptom burden, as assessed by ESSPRI and its individual domains, using adjusted multivariable model. Only participants who met the EULAR 2016 classification criteria for Sjögren’s Syndrome were included in the current study, leading to the exclusion of several cases that did not meet these updated diagnostic standards. Participants were recruited from the histopathological salivary gland archives of the Ho^∧^tel-Dieu de France, encompassing biopsies of patients examined from September 2018 to November 2024 with a Chisholm and Mason score of ≥3 (or Focus score ≥ 1). On another hand, patients were selected based on positive immunological results for anti-Sjogren’s Syndrome A (anti-SSA) and/or B (anti-SSB) antibodies conducted within the same timeframe at the same hospital. Consequently, patients were included based on either histopathology, serological markers, or a combination of both. However, final inclusion required confirmation of Sjögren’s syndrome according to the 2016 American College of Rheumatology/European Alliance of Associations for Rheumatology (ACR/EULAR) classification criteria. Final inclusion was confirmed in collaboration with each patient’s treating physician. Based on the Fisher z method for correlations, approximately 85 participants would be required to detect a moderate correlation (ρ = 0.30) with 80% power at a two-sided α = 0.05. Our final sample of 75 patients provided close to this requirement, with sufficient power for moderate-to-strong associations.

### 2.2 Questionnaires

All study participants were contacted by telephone for an interview conducted by a single investigator under the supervision of the principal investigator. Oral informed consent was obtained from all participants using a standardized, Institutional Review Board (IRB)-approved template. Participants were aware of the objectives and benefits of the study. Confidentiality was maintained during data collection and analysis. The questionnaire collected basic demographic data (age, sex, country of residence, and marital status) and disease characteristics (disease duration, date since diagnosis, treatment). Following validation by the ethics committee, The OSDI, ESSPRI, and MEDAS questionnaires, were conducted during the structured telephone interview. First, the OSDI score, a reliable instrument, was used to measure the severity of Ocular Dryness (9). OSDI effectively discriminates between normal ocular surface (0–12), mild (13–22), moderate (23–32), or severe (33 100) OD. The total score ranges from 0 to 1008. The total score is calculated according to a specific algorithm. The 12 items of the questionnaire are rated on a scale from 0 to 4, and patients assess their responses based on their perception of symptoms: 0 corresponds to “never,” 1 to “a few times,” 2 to “half of the time,” 3 to “most of the time,” and 4 to “all the time.” The final score is calculated according to this formula: sum of the scores of the questions answered by the patient × 100/(total number of questions × 4). Higher scores being correlated with greater functional impairment. In this study, the OSDI score, validated in Arabic, was used as a continuous variable. Second, the MEDAS, a 14-item questionnaire, was used to assess adherence to the MD model. This nutritional quality index has been validated in Arabic in several countries (10, 11). The telephone-administered version of MEDAS was validated for assessing the adherence to the Mediterranean diet especially for large population-based studies (11). Participants’ adherence to the MD was categorized using the MEDAS score as follows: low adherence (0–5), moderate adherence (6–9), and high adherence (10–14). These cutoffs are consistent with those used in the original validation study. In this study, the score was used as a continuous variable. And finally, we used the EULAR Sjögren’s Syndrome–Patient Reported Index (ESSPRI). Originally, scores to measure Sjögren’s syndrome activity are focused mainly on sicca symptoms. More recently, indices such as the EULAR Sjögren’s Syndrome Disease Activity Index (ESSDAI) have been developed to capture systemic disease manifestations across 12 domains, including general health, glandular involvement, joints, skin, lungs, kidneys, nerves, hematological, and immunological parameters. In contrast, ESSPRI, the score we used in our study, reflects the patient’s perspective by quantifying dryness, fatigue, and pain. Although ESSDAI and ESSPRI are complementary, they do not necessarily correlate (12). For the present study, we preferred to use a patient-reported questionnaire (ESSPRI) to focus on clinical symptomatology and because it is more accessible than certain laboratory or imaging data included in the ESSDAI, which could be missing and thereby decrease the power of the study.

### 2.3 Statistical analysis

We conducted a cross-sectional analysis of 75 participants. Continuous variables were summarized as median and interquartile IQR. The primary outcome was the association between the MEDAS and ESSPRI with its subitems (dryness, pain, and fatigue), and MEDAS and OSDI on the other hand. Because OSDI (0–100), ESSPRI (0–10), and MEDAS (0–14) are bounded scales prone to skew, we prioritized non-parametric summaries and Spearman’s rank correlations; Pearson’s r was reported as a linearity-sensitive complement. Multiple testing across MEDAS correlations with ESSPRI and its sub scores was controlled using the Benjamini–Hochberg false discovery rate. To isolate the independent effect of the Mediterranean Diet Adherence Score (MEDAS) on patient outcomes, we fitted multivariable linear regression models with ESSPRI and OSDI as dependent variables. Covariates included age, sex, smoking status, disease duration, treatment, and presence of other diseases. This approach allowed estimation of the association between MEDAS and outcomes after accounting for potential confounding factors. In the multivariable, treatment was defined specifically as systemic immunomodulatory therapy (Hydroxychloroquine, Methotrexate or Corticosteroids). Use of ocular artificial tears was analyzed as a separate descriptive characteristic but was excluded from the final regression to avoid overparameterization given the sample size. Regression coefficients (β) were reported with 95% confidence intervals (CI), and robust HC3 standard errors were applied to ensure valid inference in the presence of heteroskedasticity. All analyses were two-sided with significance set at *p* < 0.05 and were performed in R (version 4.2.3).

### 2.4 Ethics

This study was conducted in accordance with the protocol of Good Clinical Practice and the principles of the Declaration of Helsinki and was approved by the Institutional Review Board (IRB) of Saint Joseph University in Beirut (Tfem/2023/17).

## 3 Results

### 3.1 Baseline characteristics

**FIGURE 1:**
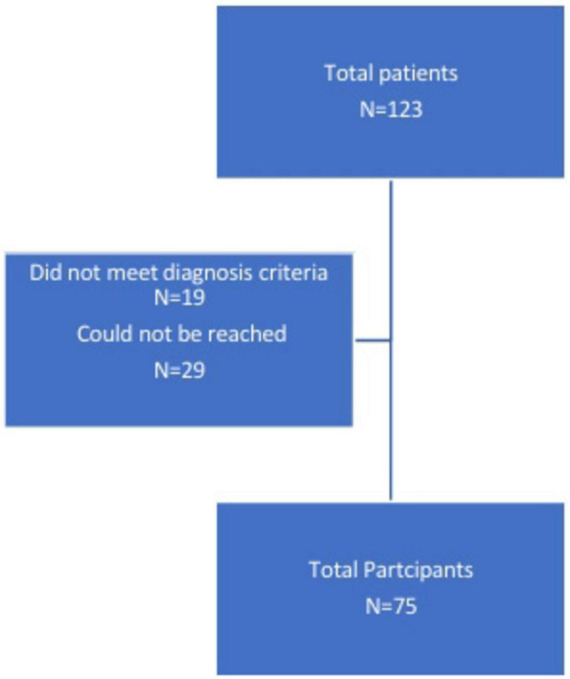
Flow chart of selected patients.

A total of 123 patients were initially recruited for the study, of whom 19 did not meet the inclusion criteria and 29 could not be reached by phone (no answer, wrong number, or out-of-service line) (Figure 1). Responses from 75 patients were included in the final analysis. The study population consisted predominantly of women (66/75, 88%), with a mean age of 50.1 ± 13.4 years (range 17–86), and age distribution did not significantly deviate from normality (Kolmogorov–Smirnov test, *p* = 0.163). Most participants were married or in a couple (57/75, 76%), while 18/75 (24%) were single, and 12/75 (16%) reported current smoking. Fifty-two percent of patients had a disease duration longer than 5 years, while 26/75 (34.7%) had disease duration <5 years. Systemic treatment (hydroxychloroquine, methotrexate, or steroids) was used by 51/75 (68%) and ocular artificial tears by 53/75 (70.7%) 0.19.6% were on methotrexate. Comorbid conditions, including autoimmune diseases (such as systemic lupus erythematosus, rheumatoid arthritis, Hashimoto’s thyroiditis, sarcoidosis and Behçet’s disease) and metabolic disorders (such as diabetes, dyslipidemia, and hypertension), were reported in 34 of 75 patients (45.3%). Patients were monitored by physicians from various specialties, including rheumatologists (46%), internal medicine doctors (31%), and dermatologists (13%), with 93% reporting regular follow-up since diagnosis. The Mediterranean Diet Adherence Screener (MEDAS) score for the cohort had a median of 8 (IQR 4.5–11.0), with 24/75 (32.0%) showing minimal adherence (0–5), 25/75 (33.3%) moderate adherence (6–9), and 26/75 (34.7%) good adherence (10–14).

### 3.2 Primary outcome: correlation between MEDAS and OSDI/MEDAS and ESSPRI and its subitems: dryness, pain, and fatigue

**FIGURE 2:**
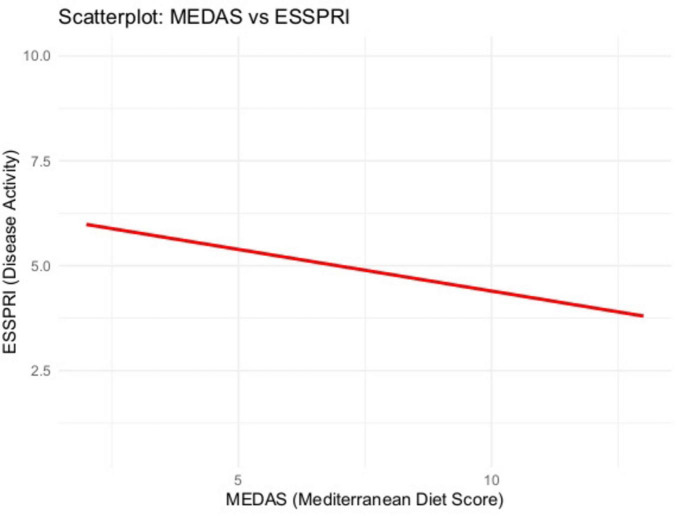
MEDAS vs. ESSPRI (scatterplot). Modest inverse association between MEDAS and global symptom burden (ESSPRI).

**FIGURE 3:**
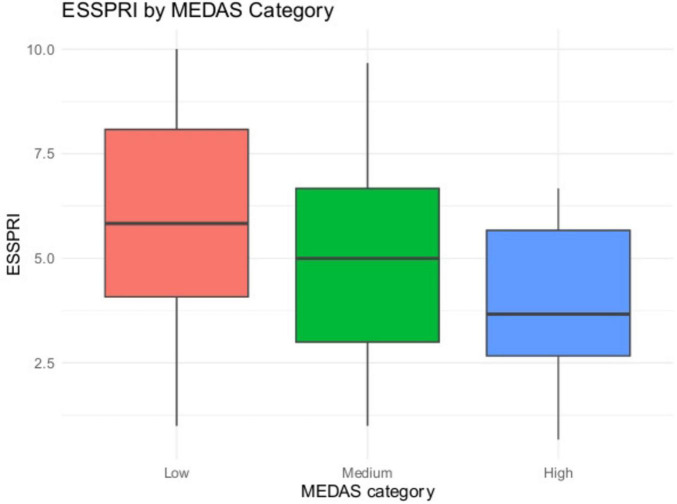
ESSPRI by MEDAS category (boxplot). Distribution of ESSPRI across low, medium, and high MEDAS categories.

**FIGURE 4:**
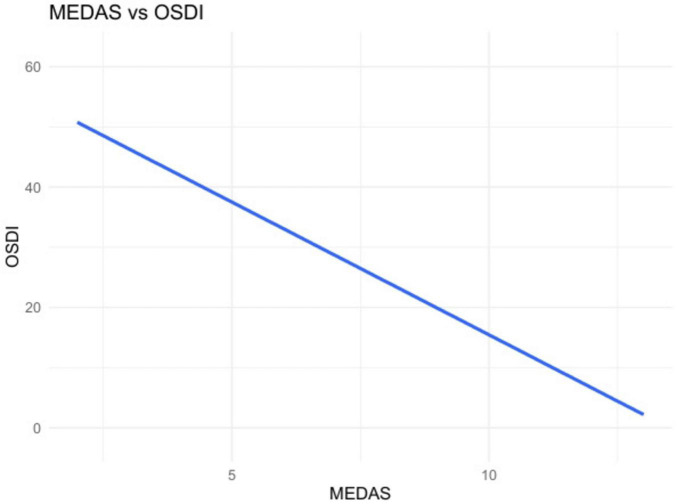
MEDAS vs. OSDI (scatterplot). Strong inverse association between diet adherence and ocular surface disease severity.

**FIGURE 5:**
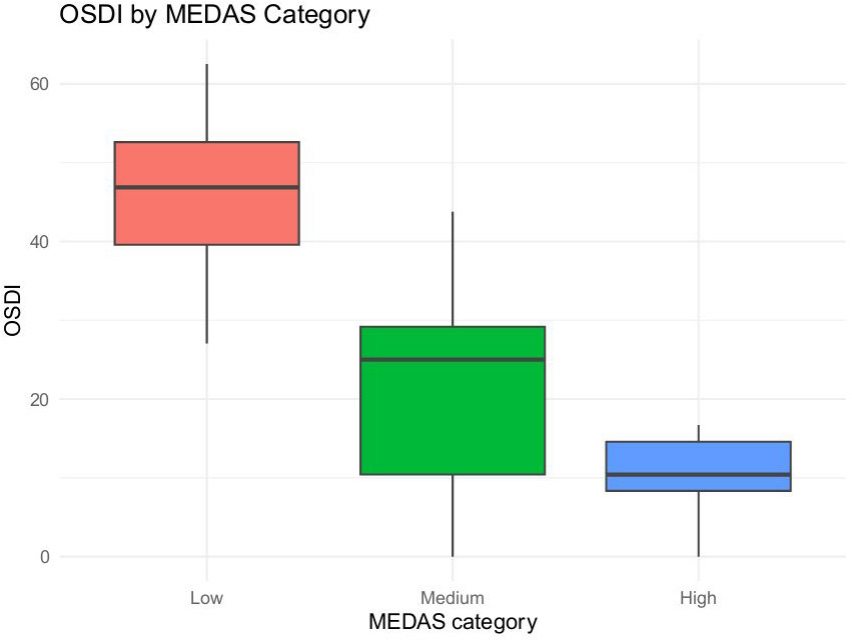
OSDI by MEDAS category (boxplot). Distribution of OSDI across low, medium, and high MEDAS categories.

We analyzed 75 patients Scores included MEDAS (Mediterranean Diet Adherence), OSDI (ocular symptoms), and ESSPRI (patient-reported dryness, fatigue, pain; total = mean of the three). In unadjusted analyses, higher Mediterranean Diet Adherence (MEDAS) was consistently associated with fewer symptoms. Specifically, MEDAS correlated negatively with global disease burden on ESSPRI (ρ = −0.258, *p* = 0.025) (Figures 2, 3) and showed a very strong inverse relationship with ocular surface disease severity on OSDI (ρ = −0.777, *p* < 0.0001) (Figures 4, 5). Similar small to-moderate inverse correlations were observed for dryness (ρ = −0.265, *p* = 0.022) and pain (ρ = −0.261, *p* = 0.024), indicating that better dietary adherence aligns with lower symptom intensity. By contrast, the association with fatigue was weak and non-significant (ρ = −0.184, *p* = 0.113). Overall, these findings suggest that greater adherence to a Mediterranean diet is linked to lower patient-reported disease activity–most prominently for ocular symptoms–while fatigue appears less diet-responsive in this cohort. To account for multiplicity across the five Spearman correlations (MEDAS vs. ESSPRI, OSDI, dryness, fatigue, pain), we controlled the false discovery rate at 5% using the Benjamini–Hochberg procedure and report FDR-adjusted *p*-values.

### 3.3 Secondary outcome: adjusted models

In multivariable linear regression models using HC3 robust standard errors (*n* = 75), greater adherence to the Mediterranean diet (MEDAS) was independently associated with lower symptom burden. For ESSPRI, MEDAS remained a significant predictor after adjustment for age, sex, smoking, disease duration, systemic treatment, and comorbidities (β = −0.22, 95% CI −0.40 to −0.04, *p* = 0.018), while none of the covariates showed independent associations. For OSDI, the association was even stronger (β = −4.58, 95% CI −5.37 to −3.79, *p* < 0.001), indicating that each 1-point increase in MEDAS corresponded to an average 4.6-point decrease in ocular surface disease severity, independent of the same covariates. Systemic treatment was positively associated with OSDI (β = +6.25, 95% CI 0.29 to 12.2, *p* = 0.043), a pattern consistent with confounding by indication (patients with more severe symptoms are more likely to receive treatment). Age, sex, smoking, disease duration, and comorbidities were not significant in the OSDI model. Collectively, these adjusted analyses reinforce that higher Mediterranean diet adherence is an independent predictor of lower patient-reported disease activity, with the largest effect observed for ocular symptoms (OSDI).

## 4 Discussion

Our study consistently shows that higher MEDAS relates to lower symptom burden: strong negative association with OSDI, and modest but significant associations with ESSPRI total and its sub scores including dryness and pain but not fatigue. These findings suggest that adopting a healthy Mediterranean diet may be associated with a decreased progression of ocular symptoms as well as pain. As a matter of fact, Mediterranean diet’s anti-inflammatory profile may reduce ocular and systemic symptoms in Sjogren’s. In this cross-sectional cohort of 75 predominantly female patients with Sjogren’s syndrome, higher adherence to a Mediterranean diet (MEDAS) was consistently associated with lower patient-reported disease burden. At the bivariate level, unadjusted model, MEDAS correlated inversely with global symptoms (ESSPRI, ρ = −0.26, *p* = 0.025), ocular surface disease severity (OSDI, ρ = −0.78, *p* < 0.0001), dryness (ρ = −0.27, *p* = 0.022) and pain (ρ = −0.26, *p* = 0.024), while fatigue showed a weak, non-significant association (ρ = −0.18, *p* = 0.113). This novel finding, unique to our study, provides new insights into the pathophysiology of fatigue in patients with Sjögren’s syndrome. This may reflect the multifactorial nature of fatigue in SS, which involves central nervous system pathways, psychological factors, and bioenergetic dysfunction rather than solely peripheral inflammation. Fatigue in primary SS is strongly associated with depression, fibromyalgia, insomnia, and other psychosocial factors independently of physiologic markers (13–15), and pain and depression are robust predictors of fatigue severity rather than inflammatory or sicca measures. Mitochondrial dysfunction and altered cellular bioenergetics have also been implicated in fatigue in Sjögren’s disease and may not be readily modified by dietary intervention alone (16). While the Mediterranean diet’s anti-inflammatory and antioxidant effects could theoretically mitigate oxidative stress, evidence directly linking nutritional patterns to fatigue relief in SS is limited, and existing nutrition studies lack robust randomized designs and fatigue-specific endpoints (17). These limitations suggest that diet may influence peripheral symptoms more effectively than central fatigue mechanisms. Future research should include targeted assessments of mitochondrial and central nervous system function, alongside fatigue-specific outcomes, to clarify whether tailored nutritional strategies can complement other therapeutic approaches for fatigue management in SS. However, since this study is cross-sectional study, a causality relationship could not be established, and other longitudinal/interventional studies are needed to determine causality. Considering the beneficial properties of key dietary components, our findings indicate that modifiable lifestyle factors–particularly diet–may serve as an adjunctive strategy in the management of SS-related ocular symptoms. Adherence to the MD, a low-cost, accessible, and non-pharmacological intervention, could provide a practical means of alleviating ocular discomfort and enhancing patients’ quality of life. These results also carry implications for patient education and clinical practice, as integrating dietary guidance into routine care may empower individuals with actionable strategies to complement conventional therapies. The simplicity and accessibility of dietary interventions further strengthen their appeal compared to pharmacological treatments, which can be costly and less well tolerated. Our findings align with our previous research. Recent cross-sectional work in pSS has similarly reported a negative association between MEDAS and OSDI scores, indicating fewer ocular symptoms in patients adhering more closely to a Mediterranean diet (8). Observational studies in Sjogren’s have also linked Mediterranean diet adherence to more favorable disease activity profiles, including lower ESSDAI and ClinESSDAI scores (18). Beyond Sjogren’s-specific cohorts, interventional studies of dry-eye patients demonstrate that adopting a Mediterranean dietary pattern (e.g., PREDIMED-PLUS) improves tear function and reduces symptoms, consistent with the strong MEDAS–OSDI association observed in our study (19). Case–control studies further suggest that individuals with higher Mediterranean diet adherence are less likely to develop pSS (20). Although there are few studies conducted on our primary outcome, the data collected reinforce the plausibility that dietary patterns exert both local (ocular) and systemic (inflammatory) benefits in Sjogren’s disease. This study has several limitations. Due to limited resources, its single center design and relatively small sample size restrict the generalizability and statistical power of the findings. Because patients were identified through a tertiary-care hospital biopsy archive, the study cohort may not be fully representative of the broader primary Sjögren’s syndrome population managed in community or outpatient rheumatology settings, potentially introducing selection bias toward more complex diagnostic cases. In addition, there are currently no published data from Lebanon describing the clinical characteristics or serologic profiles of patients with primary Sjögren’s syndrome, limiting contextual comparison. In our cohort, 71% of patients were seropositive for anti-SSA and/or anti-SSB antibodies, whereas other population-based studies, such as a Turkish cohort, have reported that approximately 15% of patients with primary Sjögren’s syndrome were seronegative for ANA, RF, anti-Ro, and anti-La autoantibodies (21). Prior work, including that of Lan et al., has shown that seronegative primary Sjögren’s syndrome represents a distinct clinical subtype characterized predominantly by exocrine gland involvement and lower B-cell activation, while seropositive disease is more frequently associated with systemic manifestations and higher disease activity (22). Uncontrolled variations in artificial tear use, the presence of heterogeneous comorbid conditions, and reliance on self-reported MEDAS scores may have introduced confounding, recall, and social desirability biases. Additionally, the reliance on the OSDI as the sole outcome measure may not fully capture ocular discomfort, given its subjective nature; objective assessments such as Schirmer’s test or Tear Break-Up Time could provide more robust insights into tear film function but this data was not always present in the medical record. The sample size of 75 provides high power for strong associations (e.g., MEDAS–OSDI), but may be underpowered to detect smaller effects, such as those with fatigue. Dietary intake was assessed using a self-reported screener (MEDAS), which may introduce recall or reporting bias. Furthermore, ESSPRI is used for quantifying symptoms reported by the patient and thus reflect his point of view. This score comprises the patient’s subjective assessment of his symptoms. ESSPRI should be complemented by the EULAR Sjogren’s Syndrome Disease Activity Index (ESSDAI). Finally, the cross-sectional design precludes evaluation of temporal changes or causal relationships between diet and symptom severity. Prospective, multicenter longitudinal studies are therefore needed to establish more robust recommendations. Despite the limitations of our study, the data suggest that Mediterranean diet adherence is strongly and consistently associated with lower ocular symptom burden and with reduced systemic symptoms in pSS particularly dryness and pain. This aligns with the anti-inflammatory and antioxidant profile of the Mediterranean diet, which may mitigate both ocular surface dysfunction and systemic autoimmune manifestations. Future research should prioritize longitudinal and interventional studies to establish causality, assess biological mediators (e.g., inflammatory cytokines, lipid mediators), and evaluate whether dietary interventions can be integrated into holistic management strategies especially for Sjogren’s disease where studies are lacking.

## 5 Conclusion

In summary, higher Mediterranean diet adherence is independently associated with lower patient-reported symptom burden in Sjogren’s syndrome, especially for ocular outcomes and pain. While causality cannot be inferred from cross-sectional data, the convergence of strong correlations, robust adjusted associations, and biological plausibility highlights diet quality as a promising target for comprehensive, patient-centered management.

## Data Availability

The original contributions presented in this study are included in this article/supplementary material, further inquiries can be directed to the corresponding author.
